# Sustainable application of rice-waste for fuels and valuable chemicals-a mini review

**DOI:** 10.3389/fchem.2023.1225073

**Published:** 2023-10-19

**Authors:** Wenwen Zhang, Xiaoyu Xu, Yongjun Yuan, Zichun Wang

**Affiliations:** ^1^ School of Food and Bioengineering, Xihua University, Chengdu, China; ^2^ Beijing Advanced Innovation Center for Soft Matter Science and Engineering, Beijing University of Chemical Technology, Beijing, China

**Keywords:** rice waste conversion, biochar, biofuel, valuable chemical, carbon/silicon-based catalyst, catalyst support

## Abstract

The global annual production of rice is over 750 million tons, and generates a huge amount of biomass waste, such as straw, husk, and bran, making rice waste an ideal feedstock for biomass conversion industries. This review focuses on the current progress in the transformation of rice waste into valuable products, including biochar, (liquid and gaseous) biofuels, valuable chemicals (sugars, furan derivatives, organic acids, and aromatic hydrocarbons), and carbon/silicon-based catalysts and catalyst supports. The challenges and future prospectives are highlighted to guide future studies in rice waste valorization for sustainable production of fuels and chemicals.

## Introduction

The excessive use of non-renewable fossil fuels causes enormous challenges, such as energy crisis and global warming (Binder and Raines, 2009; Putten et al., 2013). Biomass is the main renewable carbon resource that could replace fossil feedstocks in producing fuels and valuable chemicals (Hu et al., 2015; Sun et al., 2018; Zhang and Huber, 2018; Dodds and Gross, 2019). Compared to edible biomass such as corn, sugarcane and sweet potato, the conversion of biomass waste is promising due to non-edible, low cost and low environmental harm.

Agricultural waste, including corn stoves (leaves, stalk, and cobs), wheat straw, rice straw, rice husk, and bagasse (sugarcane), etc., is abundant with a world production of 5.1 billion tons per year in dry weight (Ho et al., 2014; Biswas et al., 2017). Particularly, the utilization of rice waste (*Oryza sativa*) is highly desired in many Asia countries such as China, India, Indonesia, Vietnam, etc., where rice is the main food crop (Wanninayake et al., 2021). In general, the rice waste mainly includes the rice husk and the rice straw. As reported in previous studies, 1 kg of harvested rice grain is accompanied by 1–1.5 kg of rice straw, and an ideal milling process can produce around 20% of rice husk (Abraham et al., 2016; Wanninayake et al., 2021; Shaheen et al., 2022). That means over 750–1,125 million tons of rice straw and around 150 million tons of rice husk are produced as rice waste every year around the world. Cellulose, hemicellulose, and lignin are the main components of rice waste (Chieng and Kuan, 2022). In rice straw, the lignocellulosic composition is 32%–47% of cellulose, 19%–27% of hemicellulose, and 5%–24% of lignin, and in rice husk, the predominant composition is 25%–35% of cellulose, 18%–21% of hemicellulose, and 26%–31% of lignin (Binod et al., 2010; Santos et al., 2016). Besides the organic components, SiO_2_ (>80%) is dominant with other oxides containing Al, Fe, Ca, Na, Mg, K, etc., that can be utilized for silica production or other materials based on these elements (Chieng and Kuan, 2022).

**FIGURE 1:**
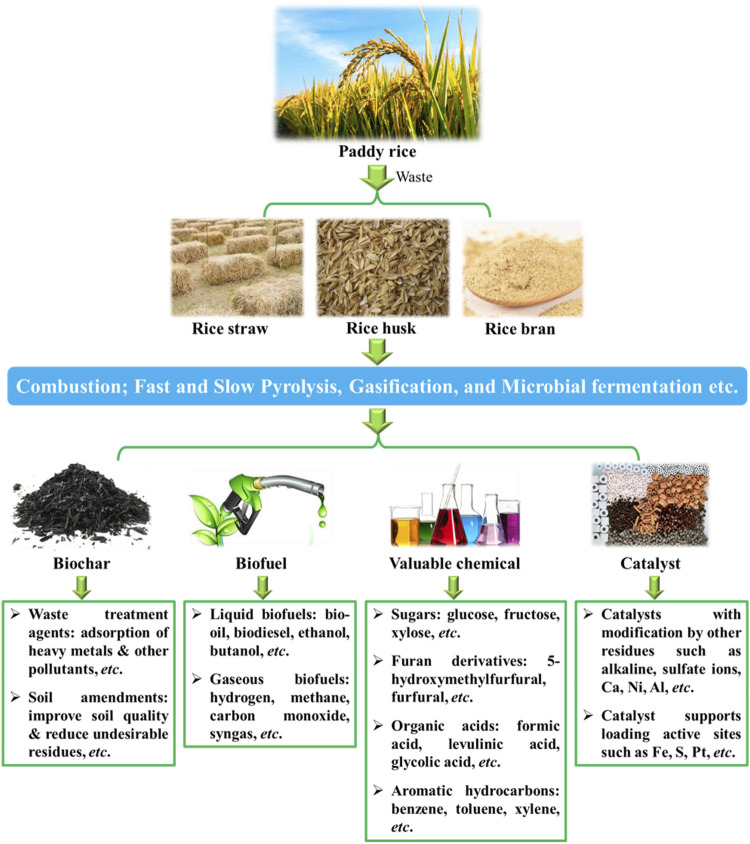
Valuable products from rice waste.

Rice waste can be converted into fuels and valuable chemicals, such as biochar, biofuels and platform molecules (e.g., glucose) (Figure 1) (Uddin et al., 2021; Shaheen et al., 2022; Shukla et al., 2022). Various methodologies have been developed for the utilization of rice waste, such as combustion, fast and slow pyrolysis, gasification and microbial fermentation. Herein, the synthesis and applications of rice waste as biochar and biofuels are firstly introduced, which act as carbon-neutral alternatives to fossil fuels. Then, the transformation of rice waste into platform chemicals is highlighted in providing renewable carbon feedstocks for sustainable chemical production with the existing industrial facilities. Furthermore, other applications of rice waste, such as in the synthesis of carbon catalyst or support materials, are also introduced. The summary of the valuable products derived from rice waste in recent years is presented in Table 1. At the end, the challenges and perspectives for the potential sustainable applications and the related chemical processes are discussed to guide future development.

**TABLE 1:** Summary of the valuable products from rice waste in recent years.

No.	Resources	Preparation routes	Products	Applications	References
1	Rice husk	Pyrolysis at 500°C and steam at 800°C under N_2_, then loading Ni and Zn	Biochar/catalysts	Steam gasification of food waste for H_2_ production	Farooq et al. (2021)
2	Huimins from rice waste conversion	Pretreated by AlCl_3_ followed by pyrolysis at 500°C–700°C	Biochar/catalysts	As Lewis acid catalysts	Xiong et al. (2021)
3	Cooked rice waste	One-pot pyrolysis of a nitrogen containing precursor of g-C_3_N_4_, cooked rice and metal salts at 500°C under N_2_	Biochar/catalysts	Low-temperature desulfurization of H_2_S	Yuan et al. (2023)
4	Rice husk	Pyrolysis at 500°C and modified with alginate under N_2_	Biochar	As waste treatment agents: adsorption of Pb in wastewater	Pham et al. (2022)
5	Rice husk	Pyrolysis at 400°C–700°C under N_2_	Biochar	As waste treatment agents: arsenic and cadmium abatement and detoxification in sediment	Zhang et al. (2020)
6	Rice straw	Pyrolysis at 400°C–700°C under N_2_	Biochar	As waste treatment agents: As and Cd abatement and detoxification in sediment	Zhang et al. (2020)
7	Rice husk	Pyrolysis at 300°C–500°C under N_2_	Biochar	As waste treatment agents: cationic dyes removal	Saravanan et al. (2021)
8	Rice straw	Impregnation-pyrolysis at 500°C under N_2_	Biochar	As waste treatment agents: crystal violet adsorption	Yi et al. (2021)
9	Rice husk	Controlled pyrolysis at 400°C–700°C under limited supply of O_2_	Biochar	As soil amendments: increase moisture content, soil microbial biomass quantity and total -C, -N, -P concentrations; enhance paddy productivity	Singh et al. (2018)
10	Rice husk	Pyrolysis at 450°C and 650°C	Biochar	As soil amendments: reducing N leaching of Calcaric Cambisols, increase soil microbial biomass and microbial activity	Bu et al. (2019)
11	Rice husk	Pyrolysis at 500°C under N_2_	Biochar	As soil amendments: decrease nitrate leaching in the soil	Mohammd et al. (2019)
12	Rice husk	Pyrolysis at 300, 450, 650°C under N_2_	Biochar	As soil amendments: reduce P sorption and increase P bioavailability in acid soil	Eduah et al. (2019)
13	Rice straw	Continuous slow pyrolysis at 500°C	Biochar	As soil amendments: immobilize heavy metals of Cd, Cu, Pb, and Zn	Lu et al. (2017)
14	Rice straw	Continuous slow pyrolysis at 500 °C	Biochar	As soil amendments: immobilize Cd and reduce the concentration of Cd in lettuce shots	Zhang et al. (2017)
15	Rice bran	Non-catalytic method using subcritical water-methanol mixture	Biofuels	Liquid biofuels: biodiesel	Zullaikah et al. (2017)
16	Rice straw	Catalytic hydroliquefaction using Ni/CeO_2_ catalysts	Biofuels	Liquid biofuels: bio-oil	Chen et al. (2018a)
17	Damaged rice grains	Presacchararification followed by simultaneous saccharification and fermentation using waste brewer’s yeast	Biofuels	Liquid biofuels: bioethanol	Mihajlovski et al. (2018)
18	Rice straw	Fermentation by *Clostridium beijerinckii* DSM 6422 pretreated by microwave-assisted hydrothermolysis	Biofuels	Liquid biofuels: biobutanol	Valles et al. (2020)
19	Rice husk	Catalytic pyrolysis via non-noble Ni-Fe catalysts supported on 5 different treated rice husk pyrolysis carbon supports	Biofuels	Gaseous biofuels: Biohydrogen	Xu et al. (2018)
20	Rice husk	Catalytic steam gasification using CeO_2_-modified Ni-CaO sorption catalysts	Biofuels	Gaseous biofuels: Biohydrogen	Zeng et al. (2022)
21	Rice husk	Solid state fermentation with *Clostridium termitidis* and *Clostridium intestinale*	Biofuels	Gaseous biofuels: Biohydrogen	Tosuner et al. (2019)
22	Rice straw	Microwave co-pyrolysis at 700°C–900°C under N_2_	Biofuels	Gaseous biofuels: Biohydrogen	Nyambura et al. (2022)
23	Rice straw	Hydrothermal pretreatment at 150°C and 210°C and subsequent anaerobic digestion	Biofuels	Gaseous biofuels: Biomethane	He et al. (2017)
24	Rice straw	Infrequently fed anaerobic digestion	Biofuels	Gaseous biofuels: Biomethane	Zealand et al. (2017)
25	Rice bran	Anaerobic digestion of de-oiled rice bran	Biofuels	Gaseous biofuels: Biomethane	Jha et al. (2020)
26	Rice straw and rice bran	Anaerobic co-digestion of rice straw and de-oiled rice bran	Biofuels	Gaseous biofuels: Biomethane	Jha et al. (2021)
27	Rice straw	Combined effect of ozonolysis and temperature hydrolysis	Biofuels	Gaseous biofuels: Biomethane	Patil et al. (2021)
28	Rice straw	Two-phase anaerobic co-digestion	Biofuels	Gaseous biofuels: Biomethane	Chen et al. (2015)
29	Rice straw	Dilute-acid hydrolysis at high temperature and pressure	Valuable chemicals	Sugars: Xylose and glucose	Karimi et al. (2006)
30	Rice straw	Mechanochemical-assisted hydrolysis and pretreated by KOH	Valuable chemicals	Sugars: Xylose and glucose	Qi et al. (2019)
31	Rice husk	Acid-hydrolysis with acid treatment	Valuable chemicals	Furan derivatives: Furfural	Bariani et al. (2020)
32	Rice husk	One-pot hydrolysis-dehydration under boron-doped biochar catalysts in ionic liquid	Valuable chemicals	Furan derivatives: Furfural and 5-hydroxymethylfurfural	Ofrasio et al. (2020)
33	Cooked rice waste	Microwave heating at 160°C under AlCl_3_ as the catalyst	Valuable chemicals	Furan derivatives: 5-Hydroxymethylfurfural	Xiong et al. (2021)
34	Rice straw	Catalytic conversion with HSO_3_-ZSM-5 zeolite catalyst under sonication	Valuable chemicals	Furan derivatives: 5-Hydroxymethylfurfural	Hoang et al. (2020)
35	Cooked rice	One-pot catalytic conversion with trivalent and tetravalent metals	Valuable chemicals	Furan derivatives: 5-Hydroxymethylfurfural	Yu et al. (2017)
36	Rice straw	Catalytic conversion with PolyE-IL catalyst	Valuable chemicals	Organic acids: Levulinic acids, formic acids and acetic acids	Ukarde and Pawar (2022)
37	Rice husk	Acid-hydrolysis with acid treatment	Valuable chemicals	Organic acids: Acetic acids, formic acids and levulinic acids	Bariani et al. (2020)
38	Rice straw	Tandem catalysis via Sn-sepiolite combined with recombinant *E. coli* whole cells harboring horse liver alcohol dehydrogenase	Valuable chemicals	Furan derivatives: furfural	Peng et al. (2019)
				Organic acids: Furoic acid	
39	Rice straw	Catalytic fast pyrolysis with hierarchical HZSM-5 treated with organosilanes	Valuable chemicals	Aromatic hydrocarbons	Zhang et al. (2018b)
40	Rice husk	Catalytic fast pyrolysis with hierarchical micro-mesoporous composite molecular sieve (MCM-41 and HZSM-5)	Valuable chemicals	Aromatic hydrocarbons and monocyclic aromatics	Li et al. (2019)
41	Rice husk	Microwave assisted catalytic fast pyrolysis with alkali-treated HZSM-5 zeolite	Valuable chemicals	Aromatic hydrocarbons: benzene, toluene, ethylbenzene and xylene	Li et al. (2020)
42	Rice straw	Catalytic pyrolysis of extracted microcrystalline cellulose with alkali modified ZSM-5	Valuable chemicals	Aromatic hydrocarbons	Nishu et al. (2021)
43	Rice straw	Catalytic pyrolysis of rice straw with Ni and alkali modified ZSM-5	Valuable chemicals	Aromatic hydrocarbons	Nishu et al. (2023)
44	Rice straw	Catalytic fast pyrolysis with hierarchical HZSM-5 modified by hexadecyl trimethyl ammonium bromide	Valuable chemicals	Aromatic hydrocarbons	Zhang et al. (2018a)
45	Rice straw	Catalytic fast pyrolysis with hierarchical HZSM-5 modified by alkali and metals	Valuable chemicals	Aromatic hydrocarbons	Chen et al. (2018b)
46	Rice straw	Pretreated with NaOH and calcined at 350°C–500°C	Catalysts	Trans-esterification of waste cooking oil for biodiesel production	Sahu (2021)
47	Rice bran	Dilute acid hydrolysis	Catalysts and feedstocks	Trans-esterification of hydrolysis rice bran	Sutanto et al. (2017)
48	Rice bran	Sulfonated carbonization at 180–250°C	Catalysts	Cellulose hydrolysis into glucose	Wataniyakul et al. (2018)
49	Rice husk	Microwave-assisted sulfonated at 200°C and fluorinated at 100°C	Catalysts	*In situ* transesterification of *Microalgae Parachlorella* kessleri biomass	Wadood et al. (2019)
50	Rice husk	Microwave-assisted acid dissolution and then loaded Fe	Catalysts	Toluene alkylation	Franco et al. (2018)
51	Rice husk	Organic acid pre-treatment and calcination at 600°C	Catalysts	As catalyst supports (silica production)	Azat et al. (2019)
52	Rice husk	Leaching with acids and then combusted at 600°C. Afterward, hydrothermal method for MCM-41 production	Catalysts	As catalyst supports (silica production), preparation for magnetic mesoporous silica MCM-41	Kamari and Ghorbani (2020)
53	Rice husk	Carbonation and loading with pyridine N, graphite N, Fe-N_x_ and thiophene S	Catalysts	Electro-catalytic reaction for oxygen reduction in Zn-air battery	Wang et al. (2022)

## Biochar

Biochar is a carbonaceous product that can be produced from the thermal decomposition of biomass, such as pyrolysis or gasification, in an oxygen-limited environment (Zou et al., 2022). Biochar derived from rice waste can feature several advantages including low cost, high carbon content, highly porous structure and rich functional groups which make it suitable for various applications in energy fuels, environmental science and new materials (Zou et al., 2022).

Biochar is utilized as excellent adsorbents for water treatment (Foong et al., 2022). To remove lead from wastewater, Pham et al. used alginate to modify biochar derived from rice husk, which provides higher surface area and more surface oxygen-contained functional groups, resulting in the uptake capacity increased from 41.2 to 112.3 mg/g, compared to the origin biochar (Pham et al., 2022). Zhang et al*.* increased the surface area of biochar through applying a higher pyrolysis temperature, which can significantly improve the absorption performance of biochar towards heavy metal, such as to reduce the concentration of arsenic and cadmium in the aqueous phase of sediment (Zhang et al., 2020). Yi et al. utilized stainless steel pickling waste liquor and rice straw to synthesize magnetic biochar (Yi et al., 2021). The adhered iron oxides (Fe_3_O_4_, Fe_2_O_3_, FeO) induced *π*-*π* interaction, hydrogen bonding and electrostatic interaction, which could remarkably enhance the adsorption capacity of biochar, e.g., up to ∼111.5 mg/g toward crystal violet in wastewater (Yi et al., 2021).

Biochar with excellent adsorption capacity can also serve in soil amendment to enhance soil organic carbon content, bulk density and microbial activity (Shaheen et al., 2022). Singh et al. prepared rice husk biochar with high surface area, rich micropores and various composition (N, P, K, Si, etc.), which can effectively increase the total N, C, and P elements in soil, regarding as a potential amendment for nutrient-deficient soils (Singh et al., 2018). Bu et al. further revealed that N leaching in biochar could be effectively reduced through synthesis rice waste biochar at high pyrolysis temperature (∼650 °C), which is attributed to the improvement of the net negative surface charge and the anion exchange capacity of biochar (Bu et al., 2019). Furthermore, biochar can immobilize heavy metals and herbicides to reduce their bioavailability in the soil (Asadi et al., 2021). For example, Lu et al. found that when 5% rice straw biochar treated the sandy loam soil, it could effectively immobilize heavy metals of Cd, Cu, Pb and Zn, thus reducing their mobility and bioavailability in contaminated soils (Lu et al., 2017). Liu et al., found that the addition of proper amount of biochar can reduce the concentration of Cd in lettuce shoots in the lightly heavy-metal-polluted greenhouse soils by immobilize the Cd in the biochar (Zhang et al., 2017).

As discussed above, the conversion of rice waste into biochar provides a sustainable production of excellent adsorbents for water treatment, the immobilization of heavy metals and herbicides, as well as soil amendment for nutrient-deficient soils. Besides increasing the nutrient elements in soil, biochar can be utilized for water and fertilizer retention and adsorption of organic matter. Moreover, the cost-effective biochar could be activated, and utilized as activated carbon in water treatment, air purification, medical, food industry and other fields, to remove pollutants, purify water and air, detoxification and so on. Most of these applications of biochar are strongly depends on its high surface area, which can be enhanced via increasing the pyrolysis temperature but is energy consuming. The preparation of biochar with high surface area at relative low temperature by optimizing synthesis conditions, such as gaseous atmosphere, could be interesting for future investigations. Decoration of biochar with surface functional groups or introducing metal active sites is promising to enhance their performance and expand their applications, such as catalysts or beyond.

## Biofuels

Biofuels are fuels derived directly or indirectly from renewable biomass (Sharma et al., 2020). Liquid biofuels are of particular interest in replacing fossil fuels, especially for transportation. The two most common types of biofuels are bioethanol and biodiesel. Other biofuels, such as methane gas and biogas are gaseous biofuels, which can be derived from the decomposition of biomass in the absence of oxygen. Through thermochemical and biotechnological pathways, rice waste can be converted into liquid (biodiesel, bio-oil, bioethanol, biobutanol, etc.) or gaseous (biohydrogen and biomethane) biofuels (Abraham et al., 2016; Gupte et al., 2022).

### Liquid biofuels

Zullaikah et al. conducted a non-catalytic method to produce biodiesel *in situ* from rice bran using a subcritical water/methanol mixture, achieving 67.4% of fatty acid methyl esters yield (200°C, 4 MPa). The high yield has been proposed by the subcritical conditions promoting the hydrolysis of rice bran oil into free fatty acids, followed by methyl-esterification (Zullaikah et al., 2017). Generally, introducing catalysts can speeds up a chemical reaction, or lowers the reaction conditions. Therefore, Chen et al. employed a series of Ni/CeO_2_ catalysts to catalyze the hydro-liquefaction of rice straw for the production of bio-oil, achieving a maximum rice straw conversion of 89.08% and a bio-oil yield of 66.7% (290°C, 2 MPa) (Chen D. et al., 2018). Compared to thermocatalytic process, microbial fermentation can produce liquid biofuels from biomass at relative milder conditions. Mihajlovski et al. processed waste rice grains through a pre-saccharification step, followed by simultaneous saccharification and fermentation using waste brewer’s yeast, achieving a bioethanol yield of 4.69% (30°C, under atmospheric pressure) (Mihajlovski et al., 2018). Valles et al. compared the efficiency of the simultaneous saccharification and fermentation (SSF) process and the hydrolysis and fermentation (SHF) process for biobutanol production from rice straw by *Clostridium beijerinckii* DSM 6422, revealing that SSF was more efficient than SHF with a biobutanol productivity of 0.114 g L^−1^h^−1^ (37°C, under atmospheric pressure) (Valles et al., 2020).

### Gaseous biofuels

Biohydrogen and biomethane are the major renewable gaseous biofuels generated from biomass. Xu et al. produced renewable biohydrogen from catalytic reforming/cracking of rice husk, using a Fe-Ni catalyst supported on the rice husk pyrolysis carbon, which provides a biohydrogen concentration of 50.5% (Xu et al., 2018). Recently, Zeng et al. prepared a bifunctional catalyst, Ce_0.7_Ni_1_Ca_5_, for the efficient production of hydrogen-rich gas from steam gasification of rice husk, achieving a higher biohydrogen concentration of 85.81 ± 0.39 vol% and yield of 35.82 ± 0.28 mmol/g _biomass_ (Zeng et al., 2022). Fermentation is a cost-effective process that often be utilized in the production of biogases in large scale. Solid-state fermentation was applied in producing biohydrogen from rice husk by Tosuner et al., and they found that the volume and yield of biohydrogen formation increased with decreasing particle size of rice husk, the highest biohydrogen formation volume was 29.26 mL under the particle size of <74 μm (yield: 5.9 mL/g _substrate_) (Tosuner et al., 2019). In producing biomethane from rice waste, optimizing the thermal pretreatment, ozonolysis induction and anaerobic co-digestion can enhance the productivity (He et al., 2017; Patil et al., 2021). In anaerobic digestion process, high thermal pretreatment temperature (e.g., 210°C) may cause longer biogasification period due to the formation of byproducts, thus He et al. found a proper thermal pretreatment temperature (150°C) achieving biomethane yield of 134 mL/(g-_Volatile solid added_) (He et al., 2017). Ozonolysis induction could help in reducing the complexity of the substrates and increasing accessibility to cellulose and hemicellulose during thermal hydrolysis rice straw (Patil et al., 2021). The co-digestion process can treat multiple waste in one facility that can keep a balanced nutrient and sufficient feedstock supply, thus improving the net biogas yield (Chen et al., 2015; Jha et al., 2021).

Biofuels is a renewable and carbon-neutral alternative to fossil fuels; however, the direct use of biofuels also have shortcomings. For example, ethanol has a lower energy density than gasoline, and the use of ethanol to partially replace gasoline creates a net energy loss. Therefore, using biofuels as feedstocks for producing bulk chemicals, such as synthesis ethylene from bioethanol dehydration, are value-added strategy (Wang et al., 2019). Hydrogen and methane are also key feedstocks in various chemical processes, such as ammonia synthesis and Fischer-Tropsch process. Exploring efficient and energy saving process for converting rice waste into biofuels is another important strategy. Using renewable energies (e.g., solar) and developing high-performance catalysts to reduce the reaction conditions are promising. Moreover, synthesis microbial by biotechnology for the efficient and selective conversion of rice wastes could promote their applications in large-scale as renewable feedstocks.

## Valuable chemicals

Besides biochar and biofuels, rice waste can also be converted into valuable carbon-based chemicals through hydrolysis, dehydration, oligomerization, decarboxylation/decarbonylation, etc. The desired products include sugars, furan derivatives, organic acids, and aromatic hydrocarbons that can be applied in the pharmaceutical, biological, medical, energy, and chemical industries.

### Sugars

Typically, sugars such as glucose, fructose, xylose, etc., are the primary products generated from the fermentation/hydrolysis of cellulose, hemicellulose, and lignin that are the main components of rice waste (Karimi et al., 2006). For the hydrolysis behavior of rice straw using dilute sulfuric acid, Karimi et al. found that in the first stage of the process, the acid-catalyzed hydrolysis of rice straw mainly depolymerized xylan into xylose with a maximum yield of 80.8% (0.5% acid, 10 min, 15 bar). Without further acid addition, the yield of glucose from glucan only increased up to 26.6% in the second stage. With the addition of more sulfuric acid (0.5%) prior to the second stage, it can achieve a glucose yield of 46.6% from glucan (3 min, 30 bar) (Karimi et al., 2006). Moreover, the pretreatment of alkali could effectively remove lignin and increase the accessibility of the substrate, and it can also change the structure of lignocellulosic biomass and make it easier to be hydrolyzed (Qi et al., 2019). Therefore, pretreatment via alkali could reduce the acid content needed for the reaction. Qi et al. found that using alkali-pretreated rice straw could significantly reduce the acid addition to 0.015 wt% HCl required for hydrolysis, and also promote the formation of glucose at a yield of 52.1% at 200°C for 60 min (un-pretreated: 13.06%) (Qi et al., 2019). Compared to the harsh conditions using liquid acids, enzyme could convert rice waste into sugars under milder conditions. For example, Park et al. have developed a facile approach to recover glucose and fructose from enzymatic saccharification of rice straw at 50°C under atmospheric pressure, resulting in yields of 40.1% for glucose and 43.5% for fructose, respectively (Park et al., 2009).

### Furan derivatives

Furan derivatives can be directly produced from rice waste as well (Bariani et al., 2020). For example, Bariani et al. synthesis furfural from sulphuric acid-catalyzed hydrolysis of rice husk, obtaining a furfural yield of 6.0 w/w% based on oven-dried rice husk weight under optimized conditions (200°C, 0.1% w/w of acid/rice husk, and 40 min) (Bariani et al., 2020). By using boron-doped biochar catalysts, Ofrasio et al. investigated the production of furan and 5-hydroxymethylfurfural (HMF) from one-pot hydrolysis-dehydration of rice straw, wheat straw or corncob, respectively, and the highest yields of furan (12%) and HMF (21%) were achieved with rice straw (Ofrasio et al., 2020). Yu et al. studied the one-pot catalysis of food waste into HMF using tri/tetravalent metal catalysts (Yu et al., 2017). SnCl_4_ offers the highest HMF yield of 22.7 wt% generated from cooked rice, which is attributed to the high activity of SnCl_4_ in the acid-catalyzed hydrolysis of starch into glucose, followed by glucose isomerization and dehydration into HMF (Yu et al., 2017; Wang et al., 2021). Further, Yu et al. reported a higher HMF yield of 35.2 mol% through Al(Ⅲ)-organic acid system in the conversion of rice waste to HMF, and the addition of maleic acid can increase the HMF selectivity by suppressing the loss of sugars and side reactions (Yu et al., 2019).

### Organic acids

The conversion of rice waste to organic acids usually starts from the degradation of rice waste to hemicellulose or oligosaccharides, then transformed into sugars. The sugars, as the platform chemicals, undergo a series of processes including isomerization, dehydration, rehydration, etc. to reach the target products of organic acids. Ukarde et al. utilized polyethyleneimine-functionalized acidic ionic liquid catalysts for the conversion of rice straw into organic acids (formic, acetic, and levulinic acids), and it was observed that the catalyst with a [HSO_4_]^−^ counter ions exhibited superior efficiency compared to other tested catalysts since it has the highest hydrophilicity and Hammett acidity (Ukarde and Pawar, 2022). Moreover, the sodium hydroxide pretreated rice straw was found to provide higher yields of levulinic acid (65.5%) and formic acid (75.8%) than untreated rice straw (levulinic acid: 49.8%; formic acid: 50.5%) (Ukarde and Pawar, 2022). Typically, these organic acids are produced along with the furan derivatives (Wanninayake et al., 2021). To convert these derivatives into acids, Peng et al. developed a one-pot chemo-enzymatic reaction system utilizing Sn-sepiolite catalyst and immobilized *E. coli* TS whole-cell biocatalyst for the efficient synthesis of furoic acids from alkali pretreated rice straw (Peng et al., 2019). The furfural generated at the initial stage under Sn-loaded sepiolite at a yield of 42.2%, followed by the completely converted into furoic acid under immobilized *E. coli* TS whole-cell biocatalyst (Peng et al., 2019).

### Aromatic hydrocarbons

Aromatic hydrocarbons, such as benzene, toluene, and xylene, are commonly produced from rice waste through the catalytic fast pyrolysis process (Zhang et al., 2018a; Li et al., 2019). H-ZSM-5 has strong acidity and heat resistance as well as excellent selective cracking catalysis and isomerisation catalytic properties, and thus, is the most frequently used catalyst in the conversion of rice wastes into aromatic hydrocarbon with high activity and selectivity (Zheng et al., 2017; Zhang et al., 2018b; Li et al., 2020). The alkali modification of ZSM-5 by potassium hydroxide could increase mesoporosity and improve the mass transfer of ZSM-5, which can significantly enhance the formation of aromatics during pyrolysis of microcrystalline cellulose extracted from rice straw, with a total aromatics relative peak area of 33.05% (0.4 M NaOH) that was higher than ZSM-5 without modification (24%) (Nishu et al., 2021). Li et al. compared the performance of HZSM-5 treated using different types of bases in the microwave-assisted catalytic fast pyrolysis of rice husk, which shows that the organic base treatment outperformed the inorganic base treatment, resulting in a 4.3% increase in monocyclic aromatic hydrocarbons and 4.6% reduction in coke formation (Li et al., 2020). Besides the alkali pretreatment, the aromatic product distribution and coke formation strongly depends on the control of hierarchical mesoporous structure (Zhang et al., 2018a; Li et al., 2019). It was found that hierarchical structure is formed by adding a certain amount of hexadecyl trimethyl ammonium bromide (0.01%) into ZSM-5, which enhanced the yield of aromatics (26.8%) and decreased the coke formation (39.2%) compared with the bare ZSM-5 (23.6%, 45.1%). (Zhang et al., 2018b). Moreover, incorporating suitable metals or other ligands into mesoporous catalysts has been shown to have an effect on the active sites of the catalysts which may influence the production of aromatic hydrocarbons. For instance, Nishu et al. synthesized Ni modified ZSM-5 for the pyrolysis of rice straw, which offers a higher selectivity to aromatics (47%) than unmodified catalyst (44%) due to the improved acidity (Nishu et al., 2023). Nevertheless, the introduction of ammonium ions in the organosilanes had a negative effect on the catalytic property of ZSM-5 that led to a lower yield (19.7%) of aromatics compared to unmodified catalyst (25.6%) due to the decreased acidity (Chen H. et al., 2018).

Sugars, furan derivatives, organic acids, aromatic hydrocarbons, etc., are generally produced from rice waste via thermocatalytic process. Most of these chemicals are platform chemicals, such as sugars, which could connect the conversion of rice wastes with the current chemical process for producing valuable chemicals. For example, glucaric acid or HMF derived from glucose can act as renewable precursors to synthesize adipic acid (Wan and Lee, 2021). The conversion efficiency strongly depends on the activity of catalysts, such as acids, alkalis, enzymes, metal oxides, etc. However, developing highly selective catalysts for specific products production is still a challenge in biomass conversion, including rice wastes, which often suffers from the lack understanding of reaction mechanisms due to the comprehensive reactions occurred.

## Catalysts

Besides the catalytic conversion of rice waste into valuable products, various catalysts can also be synthesized from rice waste to drive chemical reactions. Carbon materials can be synthesized from rice waste via hydrothermal carbonization, which can be used as catalysts or as supports for functional groups and metals (Wataniyakul et al., 2018). In some cases, rice waste derived products could also be applied as feedstocks and catalysts at the same time.

Sulfonate treatment is a common method used to introduce strong acid sites on the hydrothermal carbon. For instance, sulfonated rice husk has been utilized for the *in-situ* transesterification of microalgae at ambient temperature, obviated the steps for oil extraction after the reaction and pre-treatment of biodiesel before its application (Wadood et al., 2019). In the hydrolysis of cellulose, sulfonated hydrothermal carbon catalysts derived from defatted rice bran exhibits higher activity than the commercial Amberlyst 16 WT catalyst, with glucose and HMF yields of 19% and 46%, respectively (Wataniyakul et al., 2018).

The incorporation of active metals is another commonly used method to introduce active sites on hydrothermal carbon. Ni-loaded rice husk derived biochar was utilized as catalysts for steam gasification of food waste, exhibiting superior catalytic performance (∼68.88% gas yield) compared to Ni-loaded commercial α-alumina support (∼43.7% gas yield) (Farooq et al., 2021). This is attributed to its high reducibility, high nickel dispersion, abundant inherent K and Ca as co-catalysts, and moderate surface area (Farooq et al., 2021). Similarly, the humins generated from the reaction of starch-rich rice waste to HMF, can be valorized as biochar-supported Lewis acid catalysts after impregnation with AlCl_3_ followed by carbonization, which can catalyze glucose-to-fructose isomerization with a fructose yield of up to 14 Cmol% (Xiong et al., 2021). Moreover, Yuan et al. synthesized a N-doped biochar supported MgFe_2_O_4_ catalyst through one-pot pyrolysis of N-containing precursors, cooked rice waste, and metal salts (Yuan et al., 2023). The catalysts exhibit superior capacity (1,052.83 mg/g) for efficient low-temperature desulfurization of H_2_S with high tolerance and stability under harsh environmental conditions (Yuan et al., 2023).

The ash obtained from the combustion of rice waste contains a high concentration of silica which can be utilized as an excellent catalyst support. Franco et al. utilized microwave-assisted acid dissolution to effectively remove metal ions from rice husk, resulting in a highly pure amorphous mesoporous silica (>95%) (Franco et al., 2018). The incorporation of iron oxide in this silica framework could efficiently promote toluene alkylation with 100% selectivity toward monoalkylated products (Franco et al., 2018). Azat et al. developed an eco-friendly method for the production of high-purity silica from rice husk, utilizing dry citric acid pre-treatment and direct thermal treatment, which results in a silica yield of 20% with a purity as high as 99% (Azat et al., 2019). Kamari and Ghorbani synthesized magnetic MCM-41 with a highly ordered hexagonal structure using the silica extracted from rice husk through leaching with HCl (Kamari and Ghorbani, 2020). Benefitting from the high silica content in rice husk, the Si-Fe/S/N-RH_3_ electrocatalyst exhibited high proportions of pyridine N, graphite N and especially Fe-N_x_ and thiophene S for efficient oxygen reduction reaction, demonstrating superior activity compared to Pt/C benchmark electrocatalyst and other Fe-based electrocatalysts (Wang et al., 2022). Additionally, self-doped Si can improve the graphitization degree of the catalyst, leading to enhanced stability and methanol tolerance ability (Wang et al., 2022).

The advantage of the rice waste derived catalysts is due to their abundant and low cost. However, the purity limited their use as catalysts or supports. Seeking suitable reactions requiring low catalyst purity maybe expand their applications. Moreover, these supports consists of other elements, such as K. Developing high-performance catalysts with utilization of these elements as co-catalyts could be promising.

## Conclusion, chanlenges and future perspectives

Biomass has attracted significant attentions in the fields of biofuel and chemical conversions, as well as environmental studies. In particular, utilizing rice waste as a biomass resource holds tremendous promise for the agricultural industry. The conversion of rice waste into valuable products not only maximizes the economic benefits of agricultural biomass resources but also plays a crucial role in mitigating energy and environmental issues. This review provides a comprehensive overview of the current advancements in converting rice waste into useful products. Despite the progress made, commercializing rice waste still encounters significant challenges.

Typically, the utilization of rice waste requires multiple pretreatment processes including physical methods such as mechanical crushing and grinding, ultrasound, microwave, etc., as well as chemical methods including catalytic hydrothermal process with catalysts or enzymes and acid/alkali pretreatment. The physical pretreatment methods can reduce the size and increase the uniformity of rice waste, as well as enhance its surface area. In comparison, chemical pretreatment methods such as hydrothermal processing, acid-base treatment, and oxidation treatment are essential for degrading organic substances in rice waste. These methods break down complex organic compounds into simpler or more easily processed substances to facilitate subsequent processing steps. Despite the fact that chemical pretreatment can enhance waste availability, the processing process requires a significant amount of energy and water resources, resulting in substantial wastewater generation. This necessitates post-treatment, which increases treatment costs and environmental pressure. Additionally, high-temperature and high-pressure operating conditions pose equipment and safety risks. In order to reduce energy and water costs while addressing environmental concerns, the exploration of novel pretreatment methods utilizing regenerated high-performance catalysts or biochemical processes shows promise in achieving the pretreatment at lower costs and milder conditions.

The conversion of rice waste into valuable chemicals has been extensively studied. The current biofuel production process requires harsh reaction conditions such as high temperature, high pressure, catalyst participation and a non-oxygen environment, which is energy-intensive. Furthermore, the yields of liquid and gaseous biofuels are significantly lower than their theoretical yield. To achieve efficient biofuel production from rice waste under milder conditions, the development of high-performance catalysts and the integrated application of multiple processes could be imperative. In this case, an example of practical application could be the integration of rice waste pyrolysis into liquid fuels at a relatively low temperature and catalytic reforming of gaseous fuels for high hydrogen density. In the conversion of rice waste to chemicals, compared to solid acids, liquid acids, such as H_2_SO_4_ and HCl, can directly contact and react with rice waste to promote catalytic reactions, but this results in wastewater and environmental issues. Ionic liquid catalysts possess high accessibility and activity, making them a potential candidate for this process, however, they have not been extensively tested. Reducing the cost and enhancing reusability are pivotal factors in utilizing ionic liquids. Another viable approach could involve downsizing or breaking down waste into smaller components, thereby augmenting the surface mass transfer of waste on solid surfaces.

In practice, tailoring catalysts with high activity and stability is essential, especially in complex processes like catalytic reforming where multiple reactions occur simultaneously and rapid deactivation of catalysts is common. To enhance catalyst activity and stability, it is crucial to uncover transformation pathways and coke generation mechanisms. However, due to the complexity of reaction conditions, understanding of the reaction mechanism remains limited. Therefore, greater emphasis should be placed on investigating these reactions under *in situ* conditions to facilitate the development of high-performance catalysts for large-scale applications. With the development of efficient pretreatment techniques and an improved understanding of reaction mechanisms for designing catalysts with high activity and stability, we firmly believe that sustainable production of valuable chemicals and fuels from rice waste can be achieved, benefiting our society in the near future.
